# Robust singlet fission process in strong absorption π-expanded diketopyrrolopyrroles†

**DOI:** 10.1039/d2sc05580e

**Published:** 2022-11-16

**Authors:** Long Wang, Wenlin Jiang, Shaoting Guo, Senhao Wang, Mengfan Zhang, Zuyuan Liu, Guoliang Wang, Yanqin Miao, Lingpeng Yan, Jiang-Yang Shao, Yu-Wu Zhong, Zitong Liu, Deqing Zhang, Hongbing Fu, Jiannian Yao

**Affiliations:** Key Laboratory of Interface Science and Engineering in Advanced Materials, Ministry of Education, College of Chemistry, Taiyuan University of Technology Taiyuan 030024 China wanglong@tyut.edu.cn; Beijing National Laboratory for Molecular Sciences, CAS Key Laboratory for Organic Solids, Institute of Chemistry, Chinese Academy of Sciences Beijing 100190 China; Beijing Key Laboratory for Optical Materials and Photonic Devices, Department of Chemistry, Capital Normal University Beijing 100048 China; Beijing National Laboratory for Molecular Sciences, Key Laboratory of Photochemistry, Institute of Chemistry, Chinese Academy of Sciences Beijing 100190 China; State Key Laboratory of Applied Organic Chemistry (SKLAOC), Key Laboratory of Special Function Materials and Structure Design, College of Chemistry and Chemical Engineering, Lanzhou University Lanzhou 730000 China

## Abstract

Singlet fission (SF) has drawn tremendous attention as a multiexciton generation process that could mitigate the thermal loss and boost the efficiency of solar energy conversion. Although a SF-based solar cell with an EQE above 100% has already been fabricated successfully, the practical efficiency of the corresponding devices is plagued by the limited scope of SF materials. Therefore, it is of great importance to design and develop new SF-capable compounds aiming at practical device application. In the current contribution, *via* a π-expanded strategy, we presented a new series of robust SF chromophores based on polycyclic DPP derivatives, Ex-DPPs. Compared to conventional DPP molecules, Ex-DPPs feature strong absorption with a fivefold extinction coefficient, good molecular rigidity to effectively restrain non-radiative deactivation, and an expanded π-skeleton which endow them with well-suited intermolecular packing geometries for achieving efficient SF process. These results not only provide a new type of high-efficiency SF chromophore but also address some basic guidelines for the design of potential SF materials targeting practical light harvesting applications.

## Abstract 2

*Via* π-expanded design strategy, a new robust SF system, Ex-DPP, has been successfully developed with high extinction coefficient, good molecular rigidity and excellent SF properties.

## Graphical abstract

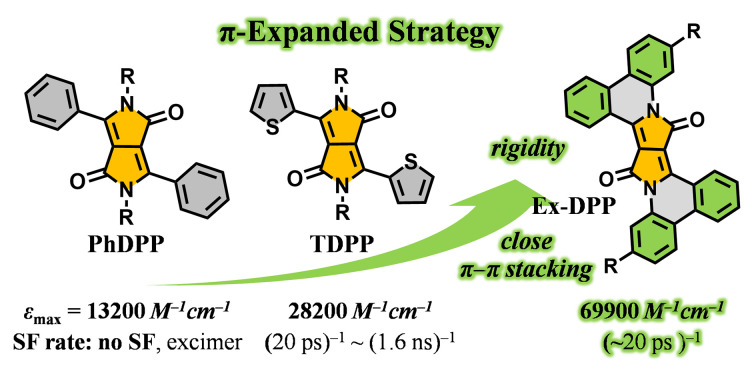

## Introduction

As a multiexciton generation pathway, singlet fission (SF) has drawn more and more attention on account of its great potential that could mitigate the thermal loss and boost the efficiency of solar cells.^1–6^ Although an SF-active solar cell with an external quantum efficiency above 100% has already been fabricated successfully,^2^ the practical power conversion efficiency of the corresponding solar cells are far behind those of conventional devices.^3,4^ The primary reason might lie in the limited scope of SF materials, which remain fairly centralized in acene and its derivatives.^3–8^ These polycyclic compounds, exemplified by tetracene and pentacene, suffer from poor chemical stability upon exposure to air and the trade-off between SF properties and energetics.^3^ For practical light harvesting applications, besides the energetic requirement that the energy of its singlet state needs to be close to two times that of its triplet state, *i.e. E*(S_1_) ≈ 2*E*(T_1_), a SF molecule should possess a large molar extinction coefficient, suitable triplet energy and good stability.^4,5,9,10^ So far, only a few of the previously reported molecules meet these criteria, such as *para*-azaquinodimethane^10^ and diimide derivatives.^11,12^ Therefore, designing and developing new SF-capable compounds aiming at practical application remains an urgent but challenging task.^3–19^

**Scheme 1:**
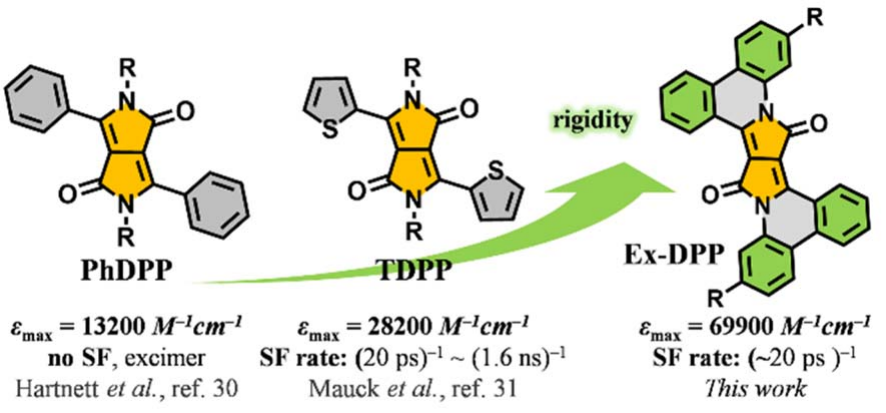
Proposed π-expanded strategy: strong absorption and good SF performances of the studied Ex-DPPs.

Diketopyrrolopyrroles (DPPs) represent a series of promising organic semiconductors in optoelectronic applications, *i.e.* organic solar cells and organic field effect transistors.^20–29^ In contrast to acene series, DPP and its derivatives have many properties, such as good structural tunability, synthetic flexibility, strong visible absorption and substantial chemical stability, which make them well-suited for SF-based light harvesting applications.^20–23,30,31^ Importantly, a DPP molecule possesses a triplet excited state energy *E*(T_1_) of ∼1.1 eV, roughly half that of its singlet state, *E*(S_1_) ∼2.2 eV, that fulfils the relationship *E*(S_1_) ≈ 2*E*(T_1_) suggesting that DPP should be an ideal SF chromophore.^32–35^ Recently, Hartnett *et al.* and Mauck *et al.* sequentially and systematically investigated the SF dynamics in a series of 3,6-diaryl-substituted DPP derivatives and presented a close correlation between the intermolecular packing geometries and SF performances in thin films.^32,33^ The phenyl substituted DPP (PhDPP) does not exhibit SF but excimer decay,^32^ while thiophen-2-yl substituted DPP (TDPP) derivatives turn out to be good SF chromophores with SF rates of up to (20 ps)^−1^ and high triplet yields (Scheme 1).^33^ However, toward practical application, DPP and its derivatives still have much room for improvement in some aspects, such as the absorption capacity and SF efficiency. In this work, *via* a π-expanded strategy, we presented a new series of robust SF chromophores based on polycyclic DPP derivatives, Ex-DPPs.^36^ Compared to conventional DPP molecules, π-expanded Ex-DPPs feature a high extinction coefficient, good molecular rigidity, and well-suited intermolecular packing geometries for achieving efficient SF process and implementing light harvesting applications (Scheme 1).

## Experimental

### Molecular synthesis and sample preparation

The studied Ex-DPP molecules, Ex-DPP1 and Ex-DPP2, were synthesized according to the previously reported routes.^36^ All reagents and solvents of the best grade available were purchased from commercial suppliers and used without further purification. Thin films were prepared by the vapor deposition method on a sapphire substrate at a rate of 0.2–0.3 Å s^−1^ under a vacuum of 1 × 10^5^ mbar. X-ray diffraction (XRD) was carried out in the reflection mode at room temperature using a 2 kW Rigaku XRD system. Grazing incidence wide-angle X-ray scattering (GIWAXS) measurements were performed on a Xeuss 3.0 SAXS/WAXS system using a Cu X-ray source (1.542 Å) with incidence angles of 0.18° at the Vacuum Interconnected Nanotech Workstation (Nano-X).

### Theoretical calculations

All the calculations were carried out with the Gaussian 16 program package.^37^ The ground state geometries were optimized at the B3LYP/6-311G* level. The vertical excitation energies of the lowest triplet and singlet states were evaluated using density functional theory (DFT) and time-dependent DFT (TDDFT) methods at the B3LYP/6-311G* level based on the optimized ground state geometries, respectively.

### Spectroscopic measurement and analysis

UV-visible absorption and fluorescence spectra were measured on a Shimadzu UV-3600 spectrometer and Hitachi F-4500 spectrophotometer, respectively. Time-resolved photoluminescence (TRPL) spectra were detected using an FLS980 spectrometer equipped with an EPL-375 picosecond pulsed diode laser (Edinburgh Instruments Ltd.). Femtosecond transient absorption (fs-TA) and nanosecond laser flash photolysis (ns-TA) measurements were all performed using the previously described instruments and experimental conditions.^38–40^ All the spectra were measured at room temperature if no further notification is given. Species-associated spectra and kinetic analyses of TA data were performed using a global fitting method with lab-written MATLAB programs. A three-state sequential kinetic model was applied to interpret TA data from thin films.^38,41^ Besides, the other analyses of the kinetic traces from TA and TRPL data were performed individually and globally using nonlinear least-square fitting to a general sum-of-exponentials function after de-convolution of the instrument response function (IRF).

## Results and discussion

### Steady state properties

**Fig. 1:**
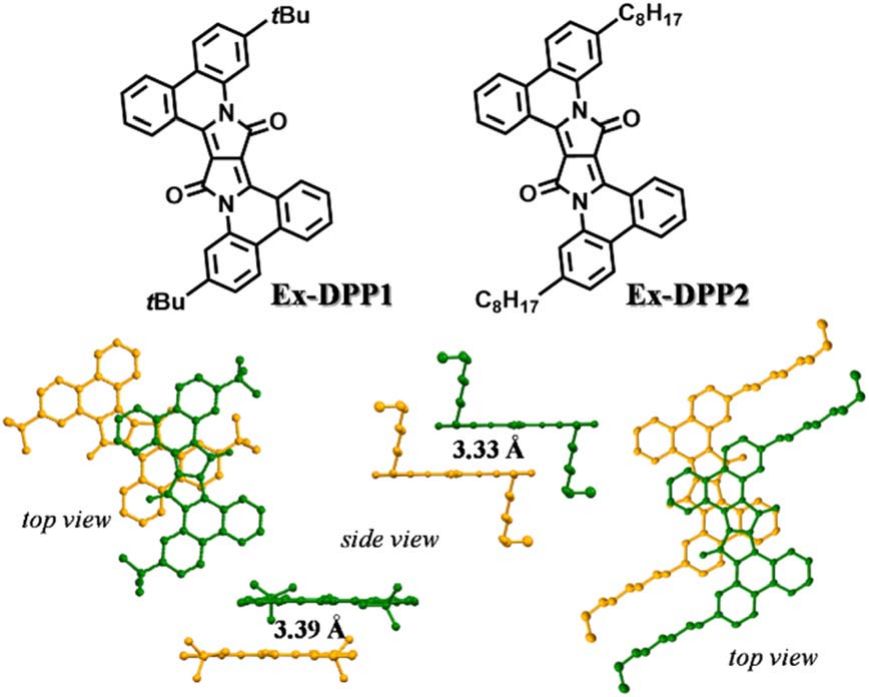
Chemical structures and molecular packing in single crystals of Ex-DPPs.

Ex-DPP molecules could be easily synthesized by the Pd(OAc)_2_-catalyzed one-step annulation of the unsubstituted DPP skeleton using the diaryliodonium salt reagent.^36^ We selected two Ex-DPP molecules as the study subjects, *i.e.*Ex-DPP1 and Ex-DPP2 with bulky *tert*-butyl and straight *n*-octyl substituents, respectively (Fig. 1). The single crystal data present that these molecules have perfectly planar molecular structures and slip-stacked intermolecular arrangements.^36^ Note that in contrast with normally slip-stacked Ex-DPP2 molecules, Ex-DPP1 molecules feature a distinctive antiparallel packing mode on account of the steric effect of the bulky *tert*-butyl substituents. Both molecules exhibits a close π–π distance of ∼3.3 Å in the most closely associated dimer unit ascribed to the perfectly planar molecular geometry in the current π-expanded polycyclic system. The previous study presented a close correlation between the intermolecular packing geometries and SF properties in a series of TDPP derivatives.^32,33^ Therefore, we are looking forward to the SF performances of these Ex-DPPs given their similar molecular packing geometries to the methyl-substituted TDPP that exhibits excellent SF properties.^33^

**Fig. 2:**
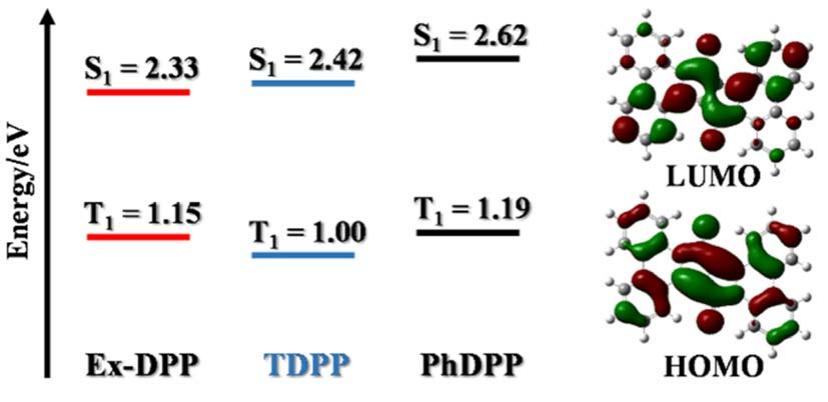
Representation of DFT calculations of singlet and triplet energy levels of DPP derivatives as well as frontier molecular orbitals of Ex-DPP.

Subsequently, the computed results from DFT calculations suggest that the lowest singlet excited state features the electronic excitation of the HOMO → LUMO, where both frontier molecular orbitals are delocalized to the entire Ex-DPP skeleton (Fig. 2). More importantly, the predicted singlet (S_1_) and triplet (T_1_) energies are 2.33 and 2.42, 2.62 and 1.15, and 1.00 and 1.19 eV for Ex-DPP, TDPP and PhDPP, respectively. Compared to the obviously exothermic condition of conventional DPPs, Ex-DPP possesses near-isoergic SF energetics of Δ*E*_SF_ = *E*(S_1_) − 2*E*(T_1_) = 0.03 eV and is even slightly endothermic taking into account the aggregation effect in thin films. The slightly endothermic condition could effectively mitigate the energy loss to a certain extent, and the triplet energy of ∼1.1 eV was deemed to be optimized for maximizing the advantages of a SF material.^4,10^ That is, the current π-expanded strategy not only does not compromise the SF energetics of these Ex-DPPs, especially triplet energy, but decreases the energy loss resulting from the excessively exothermic SF energetics of conventional DPP derivatives.

**Fig. 3:**
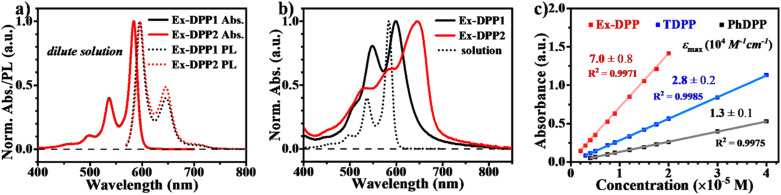
Steady state absorption and PL spectra in (a) dilute CH_2_Cl_2_ solution and (b) thin films of Ex-DPPs. (c) Extinction coefficient plot for PhDPP, TDPP and Ex-DPP (Ex-DPP1) molecules.

We then performed steady state absorption and photoluminescence (PL) measurements for these Ex-DPPs in both solution and thin films (Fig. 3a and b). In dilute solution of CH_2_Cl_2_, the spectrum of each Ex-DPP is nearly indistinguishable from each other, and presents the absorption peak maximum at 498, 537 and 585 nm (Fig. 3a). Compared to that of conventional DPPs, the absorption spectrum features vibronic fine structures with sharp characteristic peaks, which clearly prove good molecular rigidity *via* the π-expanded strategy.^42^ To reduce device thickness and mitigate problems with triplet diffusion, the SF chromophore should be equipped with a high extinction coefficient near 10^5^ M^−1^ cm^−1^.^4,5,9,10^ According to Beer's law (*A* = *εcl*, where *A* is absorbance, *ε* is the extinction coefficient, *c* is the concentration of the solution, and *l* is the pathlength), the extinction coefficient could be obtained by calculating the slope of the absorbance *vs.* concentration plot (Fig. 3c, the pathlength is 1 cm). The extinction coefficient plot displays that the Ex-DPP molecule has a value of 7.0 × 10^4^ M^−1^ cm^−1^ close to the practical criterion, which is 2.5 and 5.3 times higher than that of TDPP and PhDPP molecules, respectively.^31,33^ Steady state PL spectra of dilute solutions display small Stokes shifts and large mirror features seen in the absorption data (Fig. 3a). Similar to the conventional DPP derivatives, Ex-DPPs are highly fluorescent in dilute solution and show the fluorescence peak maximum at 596 and 645 nm with an extremely high PL quantum yield of 88% (absolute quantum yield measured using an integrating sphere) ascribed to the effective suppression of non-radiative deactivation.^31^ Therefore, the proposed π-expanded strategy not only endows these Ex-DPPs with a high extinction coefficient but also with good molecular rigidity to effectively restrain the non-radiative energy loss, which are beneficial for light harvesting applications.

In the vapour-deposited thin films, both Ex-DPP molecules feature a preferential edge-on molecular orientation as observed in the XRD and GIWAXS spectra (Fig. S4 and S5†), which is ascribed to the π-expanded planar skeleton. In these thin films, the absorption spectrum shows obvious broadening and red shifted bands relative to those in dilute solution, which indicate the strong intermolecular interactions in solid aggregates (Fig. 3b).^39,40,43^ In detail, Ex-DPP1 molecules exhibit slightly red shifted and vibrational mode-increased absorption bands, and Ex-DPP2 molecules present an obvious red shifted and enhanced absorption peak at the long wavelength. The spectral difference might be rooted in the different molecular packing geometries in these thin films as discussed in the above structural analysis from single crystals and GIWAXS data (Fig. 1 and S5†). Note that these thin films turn out to be weakly emissive and exhibit a very low PL efficiency (quantum yield < 0.01). The short-lived fluorescence excludes the accurate characterization of the PL emission signal and corresponding decay lifetime. Hence these results indicate that a highly efficient SF process might be responsible for the rapid deactivation of excited states as observed in thin films of TDPP derivatives and other molecule systems.^17,32,33,39^

### Excited state dynamics in solution

**Fig. 4:**
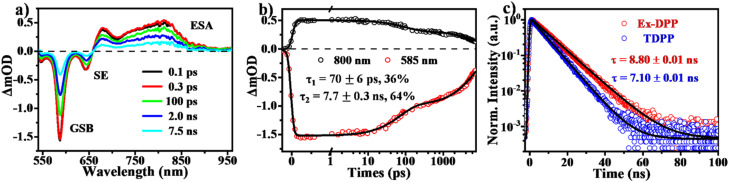
Excited state dynamics in dilute solution of the Ex-DPP molecule (Ex-DPP1). (a) Selected spectral slices and (b) kinetic decay curves from fs-TA measurements in dilute solution (535 nm excitation). (c) Transient PL decay curves of Ex-DPP (595 nm) and TDPP (565 nm) molecules in dilute solution.

Using transient absorption (TA) and PL measurements, we first investigated the excited state dynamics of these Ex-DPPs in dilute solution (Fig. 4). As shown in Fig. 4a, the TA spectra present the positive excited state absorption (ESA) bands at around 650–900 nm and the negative signals composed of ground state bleach (GSB) peaked at 585 nm and stimulated emission (SE) maximum at 645 nm. The relaxation of the ESA and the GSB signals was fit to a biexponential decay with *τ*_1_ ∼ 69 ps (36%) and *τ*_2_ = 8.4 ns (64%) (Fig. 4b), which were assigned to the lifetime of the rapid structural relaxation and radiative decay of the optically populated singlet state, respectively.^32,33^ Subsequently, the transient PL measurements (Fig. 4c) provide with a fluorescence lifetime of 8.80 ns longer than that of the TDPP molecule (7.10 ns),^31^ which further proves that the resultant molecular rigidity *via* the π-expanded strategy can effectively restrain the non-radiative loss of the excited state population. It should be noted that there is no long-lived triplet signal detected for these Ex-DPPs in dilute solution given the high fluorescence quantum yield and the inefficient intersystem crossing (ISC) pathway.^32,33^

### SF dynamics in thin films

**Fig. 5:**
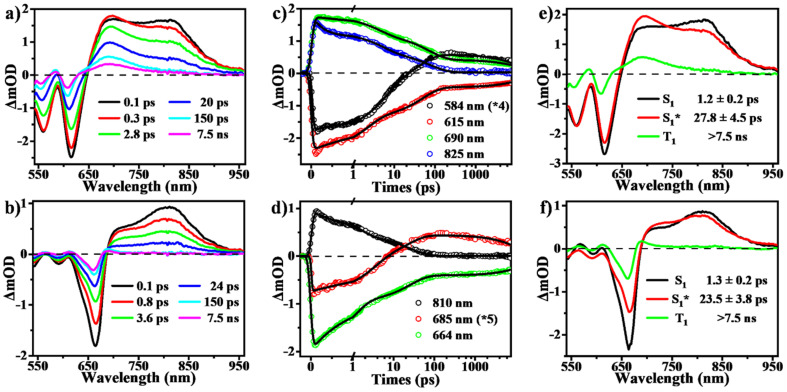
SF dynamics in thin films of Ex-DPPs. (a and b) Selected spectral slices, (c and d) kinetic decay curves and (e and f) species-associated spectra (global analysis based on a three-state kinetic model) from fs-TA measurements for Ex-DPP1 (top) and Ex-DPP2 (bottom) thin films (535 nm excitation).

**Fig. 6:**
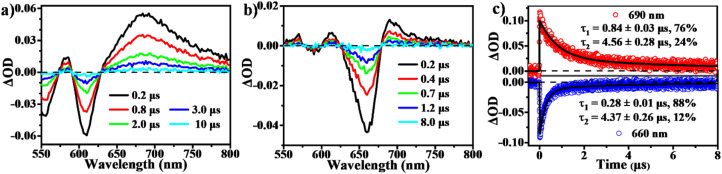
Triplet relaxation dynamics in thin films of Ex-DPPs. (a and b) Selected spectral slices and (c) kinetic decay curves from ns-TA measurements for Ex-DPP1 and Ex-DPP2 thin films (532 nm excitation).

To determine the presence of SF in the vapour-deposited thin films, fs-/ns-TA measurements were performed to give a full picture on the excited state dynamics. Compared to the solution data, the spectra from the thin films highlight their obvious evolution of TA signals and show the new long-lived excited state species with lifetimes on microsecond scales (Fig. 5 and 6). Upon photo-excitation, the spectra feature a broad ESA band in the NIR region and GSB signals at around 540–650 nm, which are assigned to the optically populated singlet state. The Ex-DPP1 film exhibits a distinctive bleach signal composed of two peaks centered at 555 and 615 nm, while the Ex-DPP2 film displays a narrow GSB at around 660 nm (Fig. 5a and b). These variations in the position of the GSB signals result from the differences in the steady state absorption bands for each Ex-DPP film (Fig. 3b). At the short time delay, the ESA signal at around 690 nm increases slightly first and then gradually attenuates together with the peak at around 820 nm (Fig. 5a and d). Over longer time scales of tens to hundreds of picoseconds, concurrent with the attenuation of the singlet ESA, new absorption bands arise at the short wavelengths overlapping with the GSB signals seriously resulting in the substantial cancellation of the GSB amplitude. The appearance of the new signal bands obviously alters the spectral line shapes suggesting the generation of a new excited state species. Such a new species turns out to be the long-lived triplet state that could persist for several microseconds (Fig. 6) as confirmed by the triplet sensitization experiments (Fig. S10†), which is consistent with the assignment from the prior reports.^32,33^ We ruled out the long-lived charge separation species given the absence of the characteristic absorption signatures of the Ex-DPP charge species in the NIR region (Fig. S11†). We also excluded the possibility of the ISC pathway because of the extremely low ISC yield in the monomer state of these Ex-DPPs. Therefore, we conclude that the long-lived triplets are undoubtedly populated by the SF process in the thin films of these Ex-DPPs. To track the evolution of the SF-formed triplet excitons, we focus on the ns-TA spectra (Fig. 6). Over time, the amplitude of the formed triplet signals decays with the biexponential kinetics of *τ*_1_ = 0.84 ± 0.03 μs (76%) and *τ*_2_ = 4.56 ± 0.28 μs (24%), and *τ*_1_ = 0.28 ± 0.01 μs (88%) and *τ*_2_ = 4.37 ± 0.26 μs (12%) in Ex-DPP1 and Ex-DPP2 thin films, respectively. Namely, the majority of the SF-formed triplets relax over hundreds of nanoseconds and the rest could persist for several microseconds. Then the triplet yields of 167% and 175% were estimated for the studied Ex-DPP1 and Ex-DPP2 thin films using the singlet depletion method previously applied in determining triplet yields in DPP systems (for details, see section 8 of the ESI†).^32,33^ Note that the singlet depletion method might partly overestimate these triplet yields.^32^

Then global analyses are performed for the TA data based on a sequential three-state kinetic model, *i.e.* S_1_ → S_1_* → T_1_. Fig. 5e and f present the species-associated spectra and corresponding time constants obtained from global analyses. The results show that for each Ex-DPP, the optically populated singlet shifts within *τ* ∼ 1 ps to a vibrationally relaxed singlet state, and then the latter transforms into the long-lived triplets within *τ* ∼ 20 ps. That is, although there are some differences in spectral line shapes, these thin films exhibit almost identical SF dynamics with a SF rate of ∼(20 ps)^−1^. We attributed this to the similar intermolecular arrangements, especially the close π–π distance of ∼3.3 Å, observed in these Ex-DPPs, which lead to the well-suited intermolecular coupling interaction for achieving an ultrafast and efficient SF process in thin films. These analyses are also in agreement with the previous SF reports by Hartnett *et al.* and Mauck *et al.*^32,33^ They presented a close correlation between the intermolecular packing geometries and SF properties in thin films of TDPP derivatives.^33^ The methyl-substituted TDPP with a π–π distance of ∼3.3 Å exhibits the most rapid and efficient SF process featuring a SF rate of (23 ps)^−1^, while the other substituted derivatives with a π–π distance more than 3.5 Å display SF dynamics that are one or two orders of magnitude slower. Therefore, the SF process in DPP skeletons turns out to be highly dependent on the intermolecular packing distances. Herein the expanded π-skeleton endows these Ex-DPPs with well-suited intermolecular packing geometries, especially a π–π distance of ∼3.3 Å, for achieving rapid and efficient SF process.^10,32,33,39,41^

Previous studies suggested that the π-expanded strategy could be successfully applied in adjusting the optical and electronic properties as well as the intermolecular interactions to improve the fluorescence emission, two-photon absorption capacity, and charge carrier mobility of organic semiconductor materials.^44–51^ Herein we address that the π-expanded strategy can be utilized to develop new practical SF materials from the following points: (I) Increasing the molecular light absorbing capacity, *i.e.* achieving a high extinction coefficient, to reduce device thickness and mitigate problems with triplet diffusion, (II) Improving the molecular rigidity to effectively restrain non-radiative loss and fully utilize high-energy photons and (III) Adjusting the molecular packing and optimizing intermolecular interaction to trigger and implement efficient SF process. Future work points to fabricating these Ex-DPPs into practical photovoltaic devices and studying the applicability of the π-expanded strategy to design new practical SF materials.

## Conclusions

In this work, we demonstrate that the π-expanded strategy can be utilized to develop potential SF materials targeting practical light harvesting applications. A new series of robust SF chromophores, Ex-DPPs, have been successfully developed. Compared to conventional DPP molecules, the current π-expanded Ex-DPPs feature strong absorption with a fivefold extinction coefficient, good molecular rigidity to effectively restrain the non-radiative deactivation, and well-suited intermolecular packing geometries for achieving efficient SF process. These results not only provide a new type of high-efficiency SF chromophore but also address some basic guidelines for the design of potential SF materials targeting practical photovoltaic applications.

## Data availability

All of the related experimental data are provided in the ESI.†

## Author contributions

L. W. conceived the project, which was supervised by L. W., D. Z., H. F. and J. Y. W. J. synthesized the compounds, which was supervised by Z. L. and D. Z. L. W. prepared samples, and conducted theoretical simulations and photophysical characterization studies including TA experiments with the assistance of S. G., S. W., M. Z. and Z. L. G. W., Y. M. and L. Y provided assistance in the GIWAXS measurements and data analysis. J. Y. S. and Y. W. Z. performed spectroelectrochemistry experiments. L. W. wrote the manuscript, and all authors participated in the data analysis and discussions.

## Conflicts of interest

There are no conflicts to declare.

## Supplementary Material

SC-013-D2SC05580E-s001
