# Supplementary material for: Robust singlet fission process in strong absorption π-expanded diketopyrrolopyrroles
Source: Chem Sci. 2022 Nov 16;13(46):13907–13. doi: 10.1039/d2sc05580e (PMC9710207; doi:10.1039/d2sc05580e)
Supplement: SC-013-D2SC05580E-s001 [file SC-013-D2SC05580E-s001.pdf]

Electronic Supplementary Material (ESI) for Chemical Science.
This journal is © The Royal Society of Chemistry 2022

Supplementary Information

Robust Singlet Fission Process in Strong Absorption π-Expanded Diketopyrrolopyrroles

Long Wang,*[a†] Wenlin Jiang,[b†] Shaoting Guo,[a] Senhao Wang,[a] Mengfan Zhang,[a] Zuyuan Liu,[a] Guoliang Wang,[a] Yanqin Miao,[a] Lingpeng Yan,[a] Jiang-Yang Shao,[d] Yu-Wu Zhong,[d] Zitong Liu,[b,e] Deqing Zhang,[b] Hongbing Fu[c] and Jiannian Yao[d]

[a] *Key Laboratory of Interface Science and Engineering in Advanced Materials, Ministry of Education, College of Chemistry, Taiyuan University of Technology, Taiyuan 030024, China. Email: wanglong@tyut.edu.cn*

[b] *Beijing National Laboratory for Molecular Sciences, CAS Key Laboratory for Organic Solids, Institute of Chemistry, Chinese Academy of Sciences, Beijing, 100190, China.*

[c] *Beijing Key Laboratory for Optical Materials and Photonic Devices, Department of Chemistry, Capital Normal University, Beijing 100048, China*

[d] *Beijing National Laboratory for Molecules Science, Key Laboratory of Photochemistry, Institute of Chemistry, Chinese Academy of Sciences, Beijing 100190, China*

[e] *State Key Laboratory of Applied Organic Chemistry (SKLAOC), Key Laboratory of Special Function Materials and Structure Design, College of Chemistry and Chemical Engineering, Lanzhou University, Lanzhou 730000, China*

Table of Contents

1. Solid Packing in Single Crystal (Figure S1-S2). **S3**

2. Theoretical Calculations (Figure S3). **S3**

3. Thin Film Characterizations (Figure S4-S5). **S4**

4. Supplemental Data for TA Measurements in Thin Films (Figure S6-S7). **S5**

5. Triplet State Relaxations (Figure S8-S9). **S6**

6. Triplet Sensitization Experiments (Figure S10). **S7**

7. Spectroelectrochemistry Measurements (Figure S11). **S8**

8. Triplet Yield Determination (Figure S12, Table S1-S2). **S9**

9. Global Analysis for TA Data from Thin Films. **S11**

10. References. **S11**

1. Solid Packing in Single Crystal.

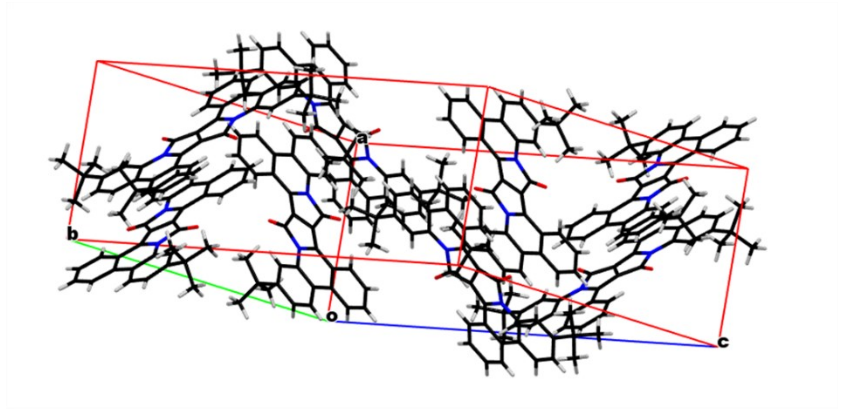

Figure S1. Solid packing in single crystal cell of **Ex-DPP1** molecules.

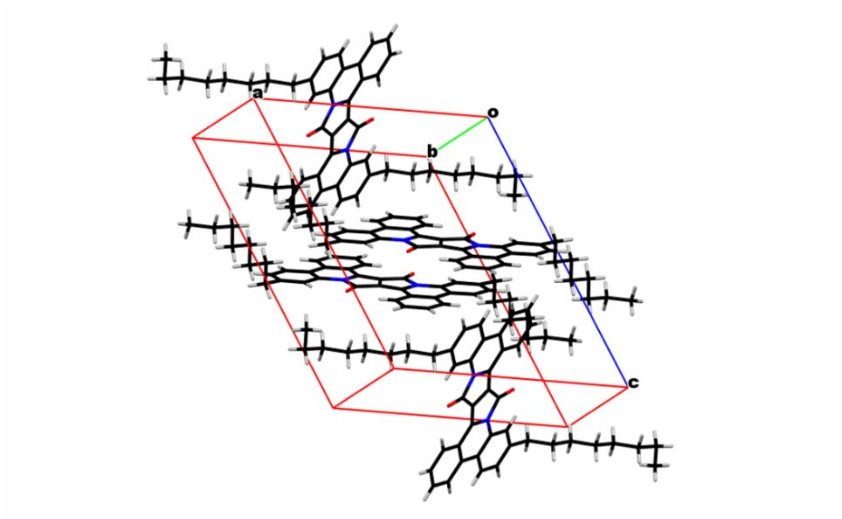

Figure S2. Solid packing in single crystal cell of **Ex-DPP2** molecules.

2. Theoretical Calculations.

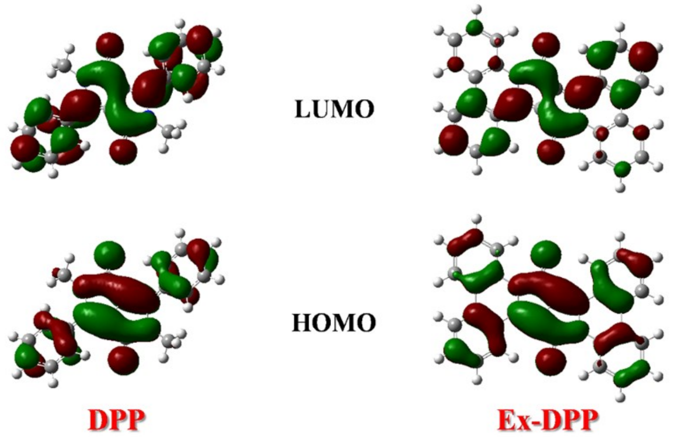

Figure S3. Frontier molecular orbitals of **PhDPP** and **Ex-DPP** based on DFT calculations.

3. Thin Film Characterizations.

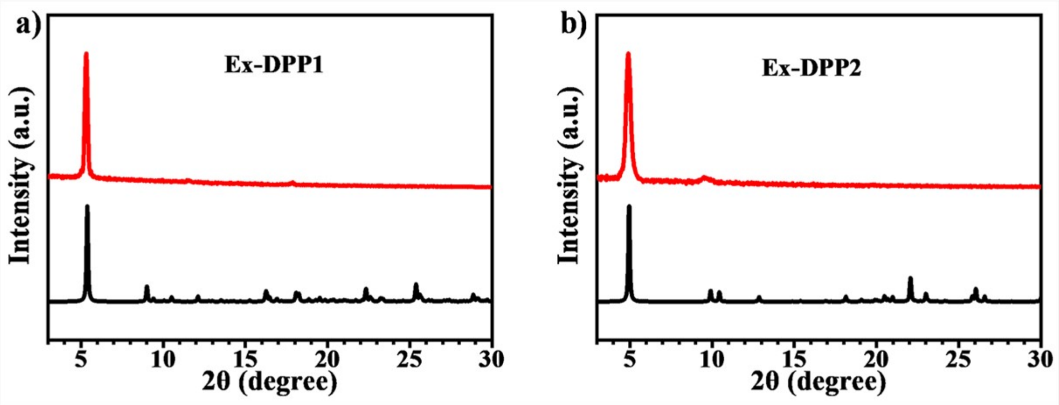

Figure S4. XRD diffractogram (red) and simulated powder pattern (black) for a) **Ex-DPP1** and b) **Ex-DPP2** thin films.

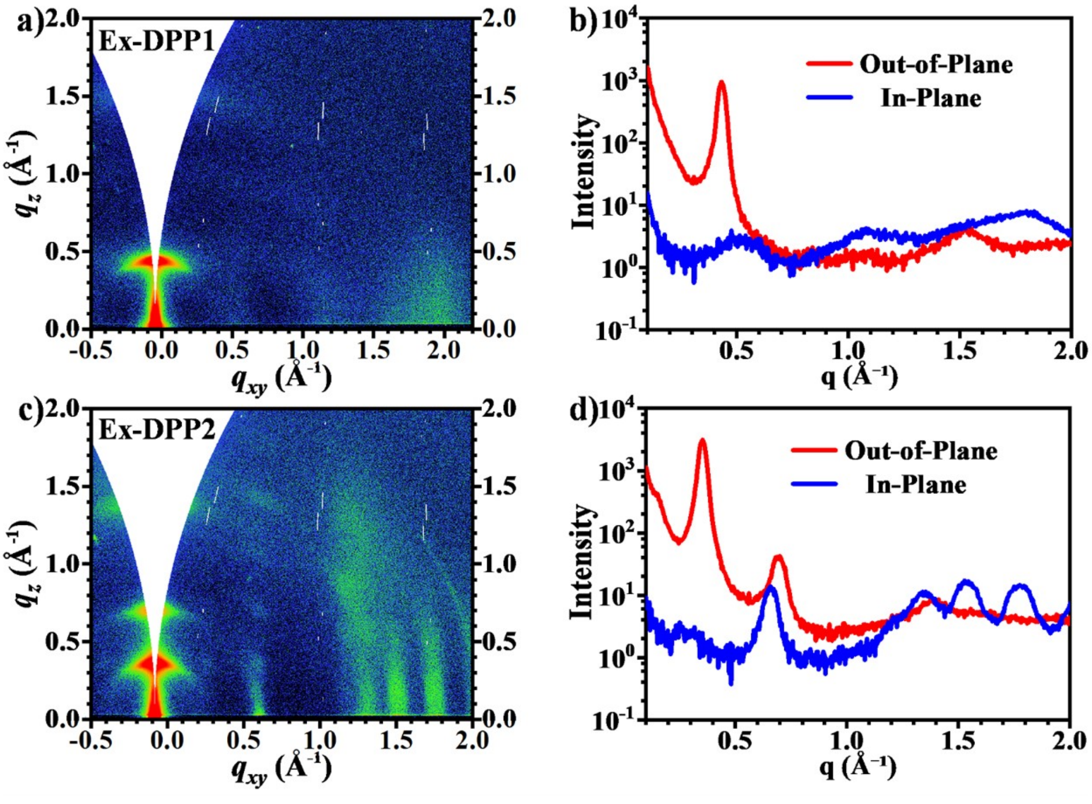

Figure S5. GIWAXS spectra and the corresponding in-plane and out-of-plane GIWAXS data for a,b) **Ex-DPP1** and c,d) **Ex-DPP2** thin films. Results show that strong lamellar diffraction peaks in the out-of-plane direction, and π-π stacking peaks in the in-plane direction are clearly observed for the thin films of two Ex-DPPs suggesting a preferential edge-on molecular orientation of Ex-DPPs in relation to the substrate. In the meantime, obvious (100) and (200) diffractions for **Ex-DPP2** film were observed at q_z = 0.35 and 0.69 Å^{-1}, respectively, indicating a higher degree of self-organization and crystallinity of linear alkyl-substituted **Ex-DPP2** molecules compared to bulky *tert*-butyl-modified **Ex-DPP1** molecules.

4. Supplemental Data for TA Measurements in Thin Films.

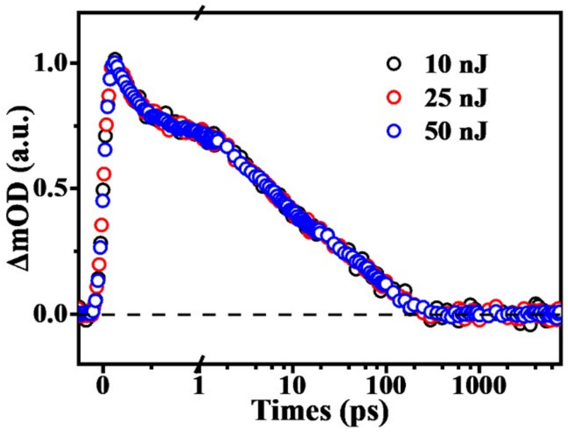

Figure S6. Normalized kinetic profiles (825 nm) for thin film (**Ex-DPP1**) upon excitation at 535 nm with different excitation densities (10, 25 and 50 nJ). Results show that the excitation density has little impact on the excited state dynamics in fs-TA measurement for thin films.

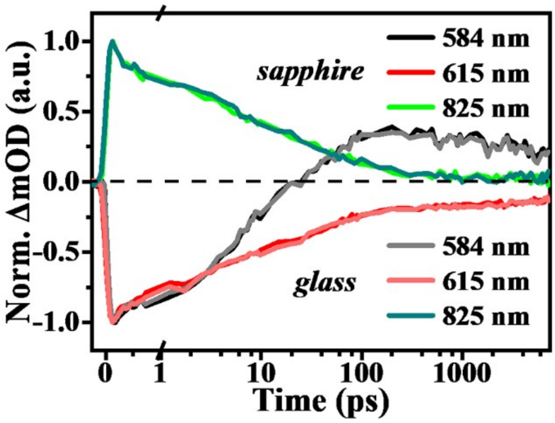

Figure S7. Normalized kinetic profiles (584, 615 and 825 nm) for thin film (**Ex-DPP1**) deposited on sapphire and glass substrates upon excitation at 535 nm. Results show that the similar decay dynamics could be observed in the corresponding selected kinetics of the TA spectra on sapphire and glass substrates.[1-3] Therefore, we suppose that no noticeable laser pump-induced heating artifacts are observed in TA measurement for these thin films.

5. Triplet State Relaxations.

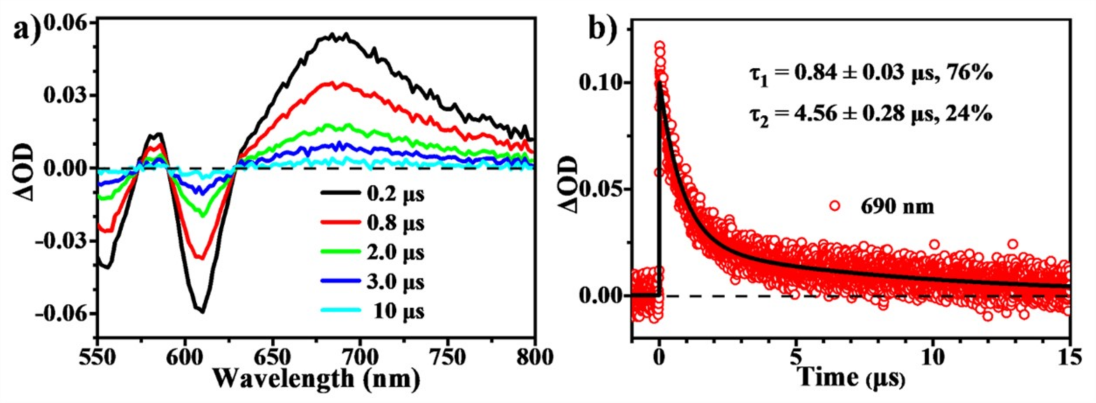

Figure S8. Long time delay ns-TA spectra and corresponding kinetics of **Ex-DPP1** thin films (excited at 532 nm).

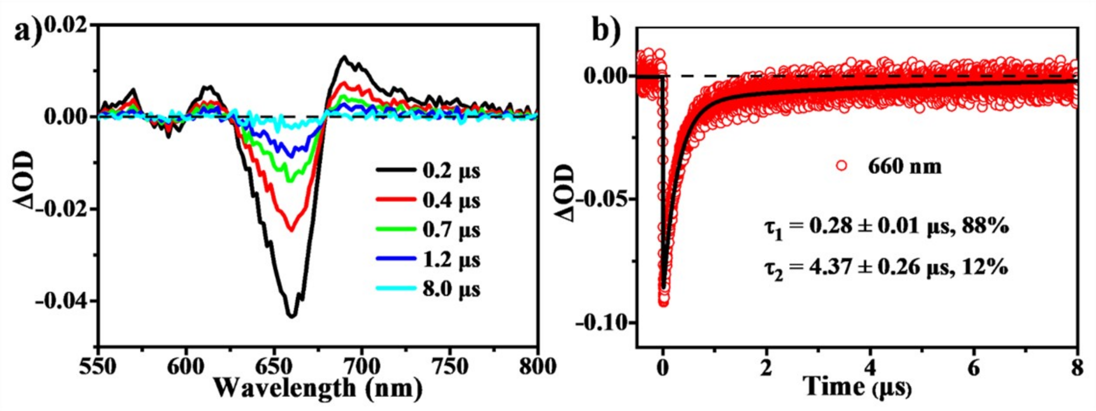

Figure S9. Long time delay ns-TA spectra and corresponding kinetics of **Ex-DPP2** thin films (excited at 532 nm).

6. Triplet Sensitization Experiments.

Triplet sensitization in solid films was performed by blending a small portion (~ 10 mol%) of $PdPc(OBu)_8$ (Pd) into PMMA or Ex-DPP thin films.[4-6] Pd was chosen as a sensitizer because it is known to undergo rapid (picosecond) intersystem crossing with quantitative triplet yield.[7,8] Then fs-TA measurements were performed by selectively exciting the Pd molecules in PMMA film or doped Ex-DPP thin film at 750 nm, where Ex-DPP molecules show negligible absorbance (**Figure S9a**, **S9b** and **S9c**). Based on the obtained TA data, we used a previously described methodology to extract the TA spectral component arising from Ex-DPP triplets (T_1).[4-6] The derived sensitized triplet spectra are in red, by taking the difference between the Pd triplet in PMMA (black) and the Ex-DPP triplets in Pd (blue) at 0.1 ns which was normalized to align the red edge (725 nm) of the PMMA and Ex-DPPs spectra (**Figure S9d** and **S9e**). The ΔOD sensitized spectrum therefore represents the overlap of the triplet ESA and the GSB signal. The sensitized triplet line shapes (red) overlap well with the experimental long-lived T_1 curves from Ex-DPP neat films (black), which confirms the dominant end species formed following photo-excitation are undoubtedly long-lived free triplets (**Figure S9f**).

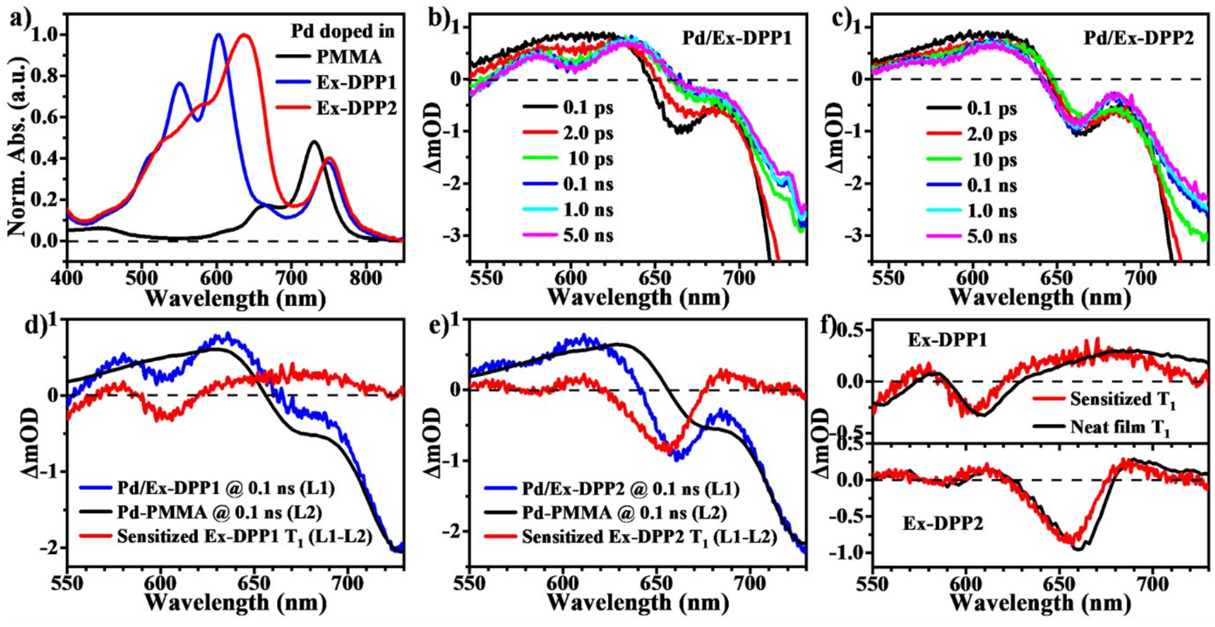

Figure S10. a) Steady state absorption and b,c) fs-TA spectra of Pd-PMMA and Pd-Ex-DPP doped films (750 nm excitation). d,e) Extraction of the sensitized triplet signatures. f) The comparison of the triplet signals from neat film (fs-TA curve at 150 ps) and sensitization experiments.

For triplet energy of the studied Ex-DPPs, the low temperature phosphorescence emission is undetectable for Ex-DPPs in both frozen solution 2-methyltetrahydrofuran and heavy metal complex doped films at 77 K. The above sensitization experiments in solid films using $PdPc(OBu)_8$ as the triplet sensitizer show that $PdPc(OBu)_8$ could sensitize the studied Ex-DPPs indicating that Ex-DPP molecule has a triplet energy less than 1.24 eV.[8] Moreover, the sensitization experiments were also performed using DiPDI as triplet sensitizer (triplet energy ~1.1 eV).[9,10] Results show that DiPDI cannot undergo triplet transfer to Ex-DPPs, which confirm that the triplet energy of Ex-DPP molecule is > 1.1 eV. That is, we could roughly estimate that the triplet energy of Ex-DPP molecule falls between 1.1 and 1.24 eV and approaches the calculated 1.15 eV.

7. Spectroelectrochemistry Measurements.

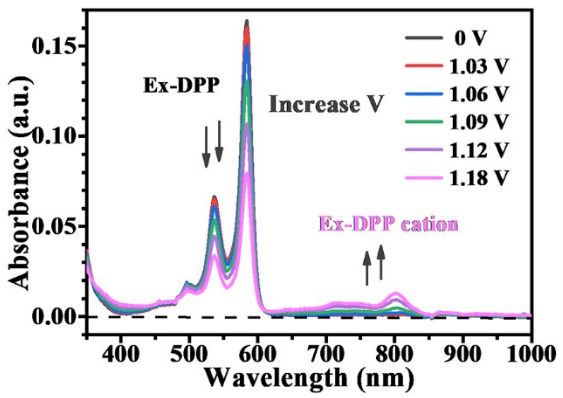

Figure S11. *In-situ* vis-NIR spectroelectrochemistry of Ex-DPP (**Ex-DPP1**) in 0.1 M Bu_4NPF_6/CH_2Cl_2. Therefore, we ruled out the long-lived charge separation species given the absence the characteristic absorption signatures of the Ex-DPP charge species in the NIR region.

8. Triplet Yield Determination.

Triplet yield was calculated with the previously reported method based on quantifying GSB signal in ns-TA data and relating this exciton density to the calculated exciton density due to the laser power, spot size, and the film thickness and absorption.[6,11,12]

These values were used to calculate an excitation density:

$$\xi = \frac{E \cdot \lambda \cdot K \cdot (1 - 10^{-A})}{l \cdot a}$$

As well as number density:

$$No.\,Density = \frac{Z}{V}$$

The ratio of which (***ξ*/*No. Density***) gives a scaling factor for the ground state absorption spectrum that produces the amount of GSB at t_0 or with 100% triplet yield. We then examine the actual bleach necessary to produce the pure triplet absorption spectrum from the ns-TA spectra and compare this to the calculated bleach. To more strictly quantify this analysis we can focus on a region where the GSB spectrum is strongly featured. As outlined by Carmichael and Hug,[13] addition of the ground state absorption spectrum to the transient trace so as to remove the minimum at this position yields a reasonable triplet-triplet absorption spectrum (**Figure S11**).

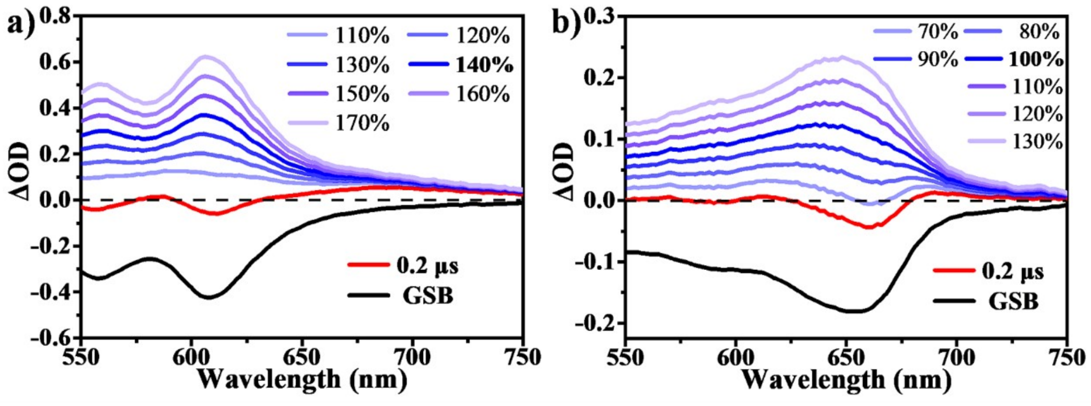

Figure S12. Triplet-triplet absorption spectra for triplet yield analysis of **Ex-DPP1** and **Ex-DPP2** thin films. Spectra are generated by addition of scaled GSB (black) to the 0.2 µs ns-TA spectrum (red).

These traces then give approximate measure of the GSB addition necessary to obtain a purely triplet-triplet spectrum and, thus, the triplet yield. The amount of GSB necessary to

produce a linear trace in this region is used as the yield. The experimental error is derived from these traces as these errors are found to be greater than the error from measurement uncertainties. As the yield is determined from a 200 ns TA trace, a portion of the triplet population has decayed as a result of triplet-triplet annihilation. To correct for this, the yield is extrapolated to t_0 using the triplet annihilation kinetics:

$$TY\% = TY\%_{200\ ns} \cdot \frac{1}{A_1 \cdot e^{-200/\tau_1} + A_2 \cdot e^{-200/\tau_2}}$$

Table S1. Laser and sample parameters for triplet yield analysis.

**Compound**	**Ex-DPP1**	**Ex-DPP2**
Excitation Energy (***E***)	0.001 *J*	0.001 *J*
Excitation Wavelength (***λ***)	532 *nm*	532 *nm*
Absorbance at excitation (A_λ)	0.256 (532 nm)	0.190 (532 nm)
Film thickness (***l***)	57.8 *nm*	57.2 *nm*
Excitation spot size (***a***)	0.785 cm^2	0.785 cm^2
Formula units/cell (**Z**)	8	2
Unit cell volume (**V**)	6477 $Å^3$	1738 $Å^3$
Absorbance at triplet (A_T)	0.638 (608 nm)	0.365 (653 nm)
Triplet annihilation kinetics (amplitudes) τ_1 (A_1) and τ_2 (A_2)	840 ns (76%)<br>4560 ns (24%)	280 ns (88%)<br>4370 ns (12%)

Table S2. Triplet yields.

**Compound**	Triplet yield at 200 ns (**TY%$_{200\ ns}$**)	**Triplet yield (TY%)**
**Ex-DPP1**	138.7%	**167.4%**
**Ex-DPP2**	95.7%	**175.4%**

9. Global Analysis for TA Data from Thin Films.

The TA data from thin film samples were fit to the following sequential three-state kinetic model (A → B → C → S_0), in which Species A, B, and C represent optically populated singlet (S_1), vibrationally relaxed singlet (S_1*), and free triplet (T_1) states, respectively:

$$\frac{d[A]}{dt} = -k_1[A]$$

$$\frac{d[B]}{dt} = k_1[A] - k_2[B]$$

$$\frac{d[C]}{dt} = k_2[B] - k_3[C]$$

The time constant $\tau_1 = 1/k_1$, $\tau_2 = 1/k_2$, and $\tau_3 = 1/k_3$.

10. References.

1. Albert-Seifried, S., Friend, R. H. Measurement of Thermal Modulation of Optical Absorption in Pump-Probe Spectroscopy of Semiconducting Polymers. *Appl. Phys. Lett.* **2011**, *98*, 223304.

2. A. Rao, Wilson, M. W. B., Albert-Seifried, S., Di Pietro, R., Friend, R. H. Photophysics of Pentacene Thin Films: The Role of Exciton Fission and Heating Effects. *Phys. Rev. B* **2011**, *84*, 195411.

3. Nichols, V. M., Broch, K., Schreiber, F., Bardeen, C. J. Excited-State Dynamics of Diindenoperylene in Liquid Solution and in Solid Films. *J. Phys. Chem. C* **2015**, *119*, 12856-12864.

4. Hartnett, P. E., Margulies, E. A., Mauck, C. M., Miller, S. A., Wu, Y., Wu, Y. L., Marks, T. J., Wasielewski, M. R., Effects of Crystal Morphology on Singlet Exciton Fission in Diketopyrrolopyrrole Thin Films. *J. Phys. Chem. B* **2016**, *120*, 1357-1366.

5. Korovina, N. V., Das, S., Nett, Z., Feng, X., Joy, J., Haiges, R., Krylov, A. I., Bradforth, S. E., Thompson, M. E., Singlet Fission in a Covalently Linked Cofacialalkynyltetracene Dimer. *J. Am. Chem. Soc.* **2016**, *138*, 617-627.

6. Mauck, C. M.; Hartnett, P. E.; Margulies, E. A.; Ma, L.; Miller, C. E.; Schatz, G. C.; Marks, T. J.; Wasielewski, M. R., Singlet Fission via an Excimer-Like Intermediate in 3,6-

Bis(Thiophen-2-yl)Diketopyrrolopyrrole Derivatives. *J. Am. Chem. Soc.* **2016**, *138*, 11749-11761.

7. Rihter, B. D., Kenney, M. E. Ford, W. E., Rodgers, M. A. J., Synthesis and Photoproperties of Diamagnetic Octabutoxyphthalocyanines with Deep Red Optical Absorbance. *J. Am. Chem. Soc.* **1990**, *112*, 8064-8070.

8. Singh-Rachford, T. N., Castellano, F. N., Pd(II) Phthalocyanine-Sensitized Triplet-Triplet Annihilation from Rubrene, *J. Phys. Chem. A* **2008**, *112,* 3550-3556.

9. Wang, L., Bai, S., Wu, Y., Liu, Y., Yao, J., Fu, H. Revealing the Nature of Singlet Fission under the Veil of Internal Conversion. *Angew. Chem. Int. Ed.* **2020**, *59*, 2003-2007.

10. Wu, Y., Zhen, Y., Ma, Y., Zheng, R., Wang, Z., Fu, H. Exceptional Intersystem Crossing in Di(perylenebisimide)s: A Structural Platform toward Photosensitizers for Singlet Oxygen Generation. *J. Phys. Chem. Lett.* **2010**, *1*, 2499-2502.

11. Eaton, S. W.; Miller, S. A.; Margulies, E. A.; Shoer, L. E.; Schaller, R. D.; Wasielewski, M. R., Singlet Exciton Fission in Thin Films of Tert-Butyl-Substituted Terrylenes. *J. Phys. Chem. A* **2015**, *119*, 4151-4161.

12. Margulies, E. A.; Logsdon, J. L.; Miller, C. E.; Ma, L.; Simonoff, E.; Young, R. M.; Schatz, G. C.; Wasielewski, M. R., Direct Observation of a Charge-Transfer State Preceding High-Yield Singlet Fission in Terrylenediimide Thin Films. *J. Am. Chem. Soc.* **2017**, *139*, 663-671.

13. Carmichael, I.; Hug, G. L., Triplet-Triplet Absorption Spectra of Organic Molecules in Condensed Phases. *J. Phys. Chem. Ref. Data* **1986**, *15*, 1-250.
